# Biological and Molecular Docking Evaluation of a Benzylisothiocyanate Semisynthetic Derivative From *Moringa oleifera* in a Pre-clinical Study of Temporomandibular Joint Pain

**DOI:** 10.3389/fnins.2022.742239

**Published:** 2022-04-25

**Authors:** Felipe Dantas Silveira, Francisco Isaac Fernandes Gomes, Danielle Rocha do Val, Hermany Capistrano Freitas, Ellen Lima de Assis, Diana Kelly Castro de Almeida, Helyson Lucas Bezerra Braz, Francisco Geraldo Barbosa, Jair Mafezoli, Marcos Reinaldo da Silva, Roberta Jeane Bezerra Jorge, Juliana Trindade Clemente-Napimoga, Deiziane Viana da Silva Costa, Gerly Anne de Castro Brito, Vicente de Paulo Teixeira Pinto, Gerardo Cristino-Filho, Mirna Marques Bezerra, Hellíada Vasconcelos Chaves

**Affiliations:** ^1^Graduate Programme in Health Sciences, Federal University of Ceará, Sobral, Brazil; ^2^Faculty of Dentistry, Federal University of Ceará, Sobral, Brazil; ^3^Graduate Programme in Biotechnology, North-Eastern Biotechnology Network, Federal University of Pernambuco, Recife, Brazil; ^4^Faculty of Medicine, Federal University of Ceará, Sobral, Brazil; ^5^Graduate Programme in Chemistry, Science Center, Federal University of Ceará, Fortaleza, Brazil; ^6^Graduate Program in Morphofunctional Sciences, Department of Morphology, Faculty of Medicine, Federal University of Ceará, Fortaleza, Brazil; ^7^Drug Research and Development Center (NPDM), Federal University of Ceará, Fortaleza, Brazil; ^8^São Leopoldo Mandic Faculty, São Leopoldo Mandic Research Institute, Campinas, Brazil; ^9^Graduate Program in Dentistry, Federal University of Ceará, Fortaleza, Brazil

**Keywords:** *Moringa oleifera*, temporomandibular joint, nociception, formalin, opioid receptors

## Abstract

**Objective:**

*Moringa oleifera* possesses multiple biological effects and the 4-[(4′-*O*-acetyl-α-L- rhamnosyloxy) benzyl] isothiocyanate accounts for them. Based on the original isothiocyanate molecule we obtained a semisynthetic derivative, named 4-[(2′,3′,4′-*O*-triacetyl-α-L-rhamnosyloxy) *N*-benzyl] hydrazine carbothioamide (MC-H) which was safe and effective in a temporomandibular joint (TMJ) inflammatory hypernociception in rats. Therefore, considering that there is still a gap in the knowledge concerning the mechanisms of action through which the MC-H effects are mediated, this study aimed to investigate the involvement of the adhesion molecules (ICAM-1, CD55), the pathways heme oxygenase-1 (HO-1) and NO/cGMP/PKG/K^+^_ATP_, and the central opioid receptors in the efficacy of the MC-H in a pre-clinical study of TMJ pain.

**Methods:**

Molecular docking studies were performed to test the binding performance of MC-H against the ten targets of interest (ICAM-1, CD55, HO-1, iNOS, soluble cGMP, cGMP-dependent protein kinase (PKG), K^+^_ATP_ channel, mu (μ), kappa (κ), and delta (δ) opioid receptors). In *in vivo* studies, male *Wistar* rats were treated with MC-H 1 μg/kg before TMJ formalin injection and nociception was evaluated. Periarticular tissues were removed to assess ICAM-1 and CD55 protein levels by Western blotting. To investigate the role of HO-1 and NO/cGMP/PKG/K^+^_ATP_ pathways, the inhibitors ZnPP-IX, aminoguanidine, ODQ, KT5823, or glibenclamide were used. To study the involvement of opioid receptors, rats were pre-treated (15 min) with an intrathecal injection of non-selective inhibitor naloxone or with CTOP, naltrindole, or norbinaltorphimine.

**Results:**

All interactions presented acceptable binding energy values (below −6.0 kcal/mol) which suggest MC-H might strongly bind to its molecular targets. MC-H reduced the protein levels of ICAM-1 and CD55 in periarticular tissues. ZnPP-IX, naloxone, CTOP, and naltrindole reversed the antinociceptive effect of MC-H.

**Conclusion:**

MC-H demonstrated antinociceptive and anti-inflammatory effects peripherally by the activation of the HO-1 pathway, as well as through inhibition of the protein levels of adhesion molecules, and centrally by μ and δ opioid receptors.

## Graphical Abstract

Semisynthetic derivative from *Moringa oleifera* on temporomandibular joint pain.

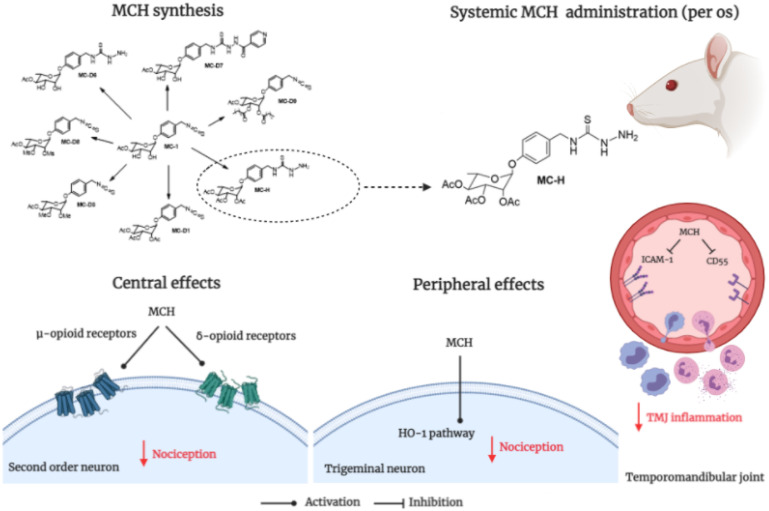

## Introduction

Temporomandibular disorders (TMD) comprise a series of conditions involving the temporomandibular joint (TMJ), masticatory muscles, and other related orofacial tissues, resulting in painful symptoms (Greene, 2010). Between 3 and 7% of the population seeks treatment for pain and dysfunction of the TMJ or related structures, affecting people aged 20-40 years and more often women (Pantoja et al., 2019; Valesan et al., 2021). TMD presents multifactorial etiology and evidence from the literature suggest that inflammation appears to be a common trigger for their onset and maintenance (Sessle, 2011, 2021; Slade et al., 2016; Costa et al., 2017). Furthermore, it has been suggested that peripheral and central neural mechanisms are involved in the orofacial inflammatory pain states (Sessle, 2011, 2021; Chichorro et al., 2017). Additionally, psychosocial components may also contribute to these conditions (Cairns, 2010; De La Torre Canales et al., 2018; Canales et al., 2019; Ettlin et al., 2021).

The TMD therapy is related to the diagnosis of each type of disfunction, and it is recommended that it initially includes less invasive and conservative approaches such as manual therapy, needling, oral splinting, exercises, acupuncture, and other physiotherapeutic techniques (Wieckiewicz et al., 2015; Dinsdale et al., 2022). Moreover, non-steroidal anti-inflammatory drugs (NSAIDs) are among the most commonly used medications to treat TMD pain (Kulkarni et al., 2020; Montinaro et al., 2022). However, evidence of the effectiveness of these drugs in the treatment of TMDs is very limited and continued use of NSAIDs is associated with several undesirable side-effects such as renal and gastrointestinal complications, raising the necessity of the development of new molecules to treat TMJ pain and inflammation (Cairns, 2010; Fine, 2013; Kulkarni et al., 2020).

*Moringa oleifera* Lam. is a tropical species whose components are largely used for medicinal purposes, among which anti-inflammatory activity has been reported (De Almeida et al., 2017; dos Santos et al., 2018). Among the different classes of metabolites isolated from the plant, thiocarbamates and isothiocyanates stand out for their biological activities (De Almeida et al., 2017). Our research group obtained seven semi-synthetic compounds from 4-(4′-*O*-acetyl-α-L-rhamnosyloxy) benzylisothiocyanate (MC-1), a secondary metabolite extracted from *M. oleifera* flowers. All derivatives and the natural product were tested for their toxicity and revealed only three semi-synthetic compounds, including *N*-[(2′,3′,4′-*O*-triacetyl-α-L-rhamnosyloxy)benzyl] hydrazinecarbothioamide (MC-H), as non-toxic at the survival rate test, biochemical, and histological analysis (dos Santos et al., 2018). Additionally, these three derivatives reduced the formalin-induced TMJ inflammatory hypernociception, while MC-H also decreased the serotonin-induced TMJ inflammation, motivating us to investigate its mechanisms of anti-nociceptive action.

Therefore, considering that there is still a gap in the knowledge concerning the mechanisms of action through which the MC-H effects are mediated, as well as given the recognized role of adhesion molecules (Ribeiro et al., 2020), regulatory systems such as heme oxygenase-1 (HO-1) (Chaves et al., 2018) and nitric oxide (NO) pathways (Chaves et al., 2011; Coura et al., 2017), and opioid analgesia during TMJ hypernociception (Silva Quinteiro et al., 2014), this study aimed to investigate the involvement of ICAM-1, CD55, the pathways HO-1 and NO/cGMP/PKG/K^+^_ATP_, and central opioid receptors in the biological effects of the MC-H in a pre-clinical study of temporomandibular joint pain.

## Materials and Methods

### Semi-Synthesis of *N*-[(2′,3′,4′-*O*-triacetyl-α-L-rhamnosyloxy) benzyl] hydrazine carbothioamide (MC-H)

The compound *N*-[(2′,3′,4′-*O*-triacetyl-α-L-rhamnosyloxy) benzyl] hydrazine carbothioamide (MC-H) was obtained and characterized according to already described by our group (dos Santos et al., 2018).

### Chemicals Solutions

This study used the following chemical solutions. Formalin (1.5%) was prepared from stock formulation (an aqueous solution of 37% of formaldehyde; Sigma Chemicals, Perth, Australia) further diluted in 0.9% NaCl (saline). Indomethacin (Indo), a non-steroidal anti-inflammatory drug used as a positive control; zinc protoporphyrin-IX (ZnPP-IX), a specific HO-1 inhibitor; aminoguanidine, a selective iNOS inhibitor; glibenclamide, an ATP-sensitive potassium channels blocker; naloxone, a non-selective opioid antagonist; Phe-Cys-Tyr-d-Trp-Orn-Thr-Pen-Thr-NH2 (CTOP), a μ-opioid receptor antagonist; naltrindole hydrochloride, a δ-opioid receptor antagonist; and norbinaltorphimine (Nor-BNI), a κ-opioid receptor antagonist. 1H-(1,2,4)-oxadiazole(4,2-a) quinoxaline-1-one (ODQ; an inhibitor of soluble guanylate cyclase enzyme) was obtained from Tocris Cookson, Ballwin, MO, United States. Indole [2,3-a] pyrrolo[3,4-c] carbazole aglycone (KT5823; an inhibitor of protein kinase G) was obtained from Calbiochem (San Diego, CA). 1H-(1,2,4) -oxadiazole (4,2-a) quinoxaline-1-one (ODQ) zinc protoporphyrin-IX and KT5823 were dissolved in dimethyl sulfoxide (DMSO) (Sigma, St. Louis, MO, United States) and resuspended in saline to minimize the final concentration of DMSO (max. 0.5%). The glibenclamide was dissolved in 2% Tween 80 and resuspended in saline. Morphine sulfate (Dimorf^®^) was purchased from Cristália (Itapira, SP, Brazil).

### Computational Simulations of the MC-H - Receptor Interactions

The molecular structure of MC-H was 3D-modeled using software Avogadro 1.1.2 (Hanwell et al., 2012) and geometrically optimized by using the density functional theory (DFT) with correlation functional B3LYP and base 6-31G(d) from software GAMESS (Barca et al., 2020). The protein structures of ICAM-1 (PDB: 1IC1), CD55 (PDB: 1OK2), HO-1 (PDB: 1N3O), iNOS (PDB: 3NQS), soluble cGMP (PDB: 3OD0), cGMP-dependent protein kinase (PKG) (PDB: 6C0T), K^+^_ATP_ channel (PDB: 6C3P), as well as of mu (μ) (PDB: 4DK1), kappa (k) (PDB: 4DJH) and delta (δ) (PDB: 6PT3) opioid receptors were obtained from Protein Data Bank^1^. All structures were resolved by X-ray diffraction at a resolution of 1.20 – 3.10 Å. The docking positions were based on the native ligand for each molecular target using Web Server Computed Atlas of Surface Topography of proteins – CASTp^2^. For molecular docking simulations AutoDock Tools (ADT) v4.2 was used to prepare targets and ligands (Pettersen et al., 2021) and AutoDock Vina 1.1.2 for calculations (Trott and Olson, 2010). Binding affinity and residue interactions were used to determine the best molecular interactions. The results were visualized by ADT, Discovery Studio v4.5 (BIOVIA, Dassault Systèmes, BIOVIA Workbook), and UCSF Chimera X (Pettersen et al., 2021).

### Animals

Male *Wistar* rats (180-240 g), provided by the Animal Care Unit of the Federal University of Ceará (UFC - Fortaleza, Brazil), were randomly housed in appropriate plastic cages at 23 ± 2°C with a 12-h light-dark cycle (light from 06:00 AM to 6:00 PM), and access to water and food *ad libitum*. In order to avoid any bias from the fluctuation of estrogen during the menstrual cycle, male rats were used in this sequence of experiment. Rats were allotted to groups of five animals (*n* = 5) and handled with special care to avoid environmental disturbances. The study was conducted under the International Association for the Study of Pain (IASP) guidelines on the use of laboratory animals for investigations of experimental pain in conscious animals (Zimmermann, 1983). The local ethics committee approved all the experiments under registration number 03/2015.

### Induction of Temporomandibular Joint Hypernociception by Formalin

The testing procedures took place between 9:00 AM and 5:00 PM in a controlled quiet room maintained at 23 ± 2°C (Rosland, 1991). Before the experiments, to provide an acclimatization process to experimental manipulation, each rat was briefly handled each day for 7 days (Macedo et al., 2016). Rats were individually placed into a mirrored-wood chamber (30 cm × 30 cm × 30 cm) with glass at the front side for 15 min to minimize stress. Each animal received 50 μl of formalin (1.5%) or saline (0.9%) (Sham group) into the left TMJ after brief anesthesia with isoflurane (3%, 30 s). The TMJ injection was performed with a 30-gauge needle connected to a 50-μL Hamilton syringe according to the method previously described (Roveroni et al., 2001). Subsequently, rats regained consciousness after approximately 30 s and then they were placed back in the test chamber for a 45-min observation period. A trained examiner evaluated the nociceptive responses which were the cumulative total number of seconds that the animal spent rubbing the orofacial region asymmetrically with the ipsilateral fore or hind paw and the number of head flinches. All experiments used double-blind masking in which the person who injected the solutions was not the same person who assessed the behavioral responses (Roveroni et al., 2001; Clemente et al., 2004). Previously, our research group showed that MC-H (1 μg/kg; *po*) was the most efficient dose in the experimental model of TMJ hypernociception (dos Santos et al., 2018). Thus, to elucidate the mechanism of action of this semisynthetic derivative, this dose was used in the following assays.

### Effects of the MC-H on the Levels of Intercellular Adhesion Molecule (ICAM-1) and Decay-Accelerating Factor (CD55) on Formalin-Induced TMJ Hypernociception

To evaluate ICAM-1 and CD55 protein levels in the TMJ periarticular tissues, rats were divided into three groups (*n* = 5): sham (treated with oral saline and after 1 h received intra-articular saline injection), MC-H (1 μg/kg treatment and 1 h after intra-articular 1.5% formalin injection) and formalin (treated with oral saline and 1 h after intra-articular 1.5% formalin injection). TMJ periarticular tissue samples were macerated in a polytron homogenizer^®^(Thomas Scientific). The product of this process was mixed in 500 μl of RIPA buffer (25 mM Tris-HCl pH 7.6; 150 mM NaCl; 5 mM EDTA; 1% NP-40; 1% Triton-X-100; 1% sodium deoxycholate; 0.1% SDS) and protease inhibitors. The samples were then vortexed for 30 seconds, this process was repeated 2 more times every 10 minutes and then the samples were centrifuged (17 min, 4°C, 13000 rpm). After discarding the pellet, the total proteins were dosed by the bicinchoninic acid method and performed as described by the manufacturer (Thermo Scientific, United States). For the western blotting procedure, equal amounts of proteins (20 μg) from the TMJ periarticular tissue were treated with sample buffer (BioRad, United States 65.8 mM Tris-HCl, pH 6.8, 26.3% glycerol, 2.1% SDS, 0.01% bromophenol blue) and β-mercaptoethanol (BioRad, United States), vortexing for 10 s, heating in the water bath (95°C, 5 min). Thereafter, samples were centrifuged (10000 rpm, 4°C, 30 s) and then vertical protein electrophoresis on a 10%-polyacrylamide gel (SDS-PAGE) was performed at 60 V for the first 15 min and 120 V for the remaining time, in which running buffer (25 mM Tris; 192 mM glycine; 1% SDS) was used. Transferal of the gel proteins to PVDF membranes (BioRad, United States, polyvinylidene fluoride) was performed by electrophoresis at 100 V for two hours in the transfer buffer (25 mM Tris, 192 mM glycine, 20% methanol). After this step, membranes were blocked to avoid non-specific binding with 5% BSA (Sigma-Aldrich, United States) diluted in Tris-HCl buffer supplemented with Tween 20 (TBST-20 mM Tris pH 7.5, 150 mM NaCl, 0.1% Tween 20). The samples were then incubated overnight at 4°C under constant stirring with anti-ICAM-1 (Abcam, 1:100), anti-CD55 (Santa Cruz Biotechnology, 1:100), or anti-α-tubulin antibodies (Millipore, EP1123Y, 1: 500), used as a control, all diluted in 1%-BSA in TBST. Membranes were incubated with the HRP-goat anti-rabbit secondary antibodies (Invitrogen, 656120, 1:1000) or HRP-rabbit anti-goat (Invitrogen, A16142, 1:1000) for two hours at room temperature. Finally, the bands were recognized by their respective antibodies, following a chemiluminescence procedure (BioRad, United States, Clarity western ECL blotting substrate), being visualized with the system ChemiDoc XRS (BioRad, United States). The band densities were measured by ImageJ software (NIH, Bethesda, MD, United States).

### Involvement of the HO-1 Pathway on the MC-H-induced Antinociception

Animals were pre-treated (30 min) with zinc protoporphyrin-IX (ZnPP-IX) (3 mg/kg, *sc*), followed by administration of the MC-H (1 μg/kg; *po*) 1 h before intra-TMJ injection of formalin (1.5%, 50 μL/TMJ). The sham group received the vehicle of the ZnPP-IX (*sc*) followed by administration of the MC-H vehicle (*po*) 1 h before intra-TMJ injection of saline (50 μL/TMJ). Behavioral nociception responses were evaluated for a 45-min observation period.

### Role of NO/cGMP/PKG/ATP-Sensitive Potassium Channel Pathway in the MC-H-Induced Antinociception

Rats were pre-treated (15 min) with aminoguanidine (30 mg/kg; *ip*), a selective inhibitor of inducible nitric oxide synthase (iNOS), ODQ (5 mg/kg; *sc*), an inhibitor of soluble guanylate cyclase enzyme (sGC) what generates cyclic guanosine monophosphate (cGMP), KT5823 (4 mg/kg, *sc*), an inhibitor of protein kinase G (PKG), or glibenclamide (10 mg/kg; *ip*), an ATP-sensitive potassium channel blocker (K^+^_ATP_) followed by the MC-H (1 μg/kg; *po*) administration 1 h before the intra-TMJ injection of 1.5% formalin (50 μl/TMJ). The sham group received a pre-treatment (15 min) of the vehicle of aminoguanidine (*ip*), ODQ (*sc*), KT5823 (*sc*) or glibenclamide (*ip*), followed by the administration of the MC-H vehicle (*po*) 1 h before the intra-TMJ injection of saline (50 μL/TMJ). Behavioral nociception responses were evaluated for a 45-min period of observation.

### Effects of the Central Opioid Receptor Antagonists (μ, δ, κ) on the MC-H-Induced Antinociception

In another series of experiments, rats received briefly isoflurane anesthesia and took an intrathecal injection of naloxone (15 μg/10 μL), a non-specific opioid antagonist, as previously described (Fischer et al., 2005). To evaluate the participation of specific opioid receptors, it was used CTOP (10 μg/μl), naltrindole (30 μg/10 μL) or norbinaltorphimine (45 μg/10 μL), μ, δ, and κ selective opioids receptors antagonists, respectively. After 15 min after naloxone, CTOP, naltrindole, or norbinaltorphimine intrathecal administration, rats received MC-H (1 μg/kg). One hour after MC-H administration, the animals received an intra-TMJ injection of formalin (1.5%, 50 μl). The sham group received intrathecal injection of the vehicle of naloxone, CTOP, naltrindole or norbinaltorphimine. After 15 minutes, they received the MC-H vehicle (*po*) 1 h before the intra-TMJ injection of saline (50 μL/TMJ). Behavioral nociception responses were evaluated for a 45-min period observation following formalin injection.

### Statistical Analysis

Normality was evaluated by the Shapiro-Wilk test. Student’s *t*-test or one-way ANOVA was used to analyze associations between the variables. The *post hoc* test for ANOVA was chosen according to the result of the homogeneity of variances (Levene’s test). For homoscedastic variances Tukey’s test was applied, while for heteroscedastic variances the Games-Howell test was used. Results are presented as means ± SEM or mean ± SD for parametric. All tests were performed using SPSS 20.0 (SPSS Inc., Chicago, IL, United States) program for Windows. All graphs were made with Graph Pad Prism 6 (Graph Pad Prism software, La Jolla, CA, United States) software for Windows. Probability level (*p*-value) < 0.05 was assumed.

## Results

### Computational Simulations of MC-H-Receptor Interactions

**FIGURE 1:**
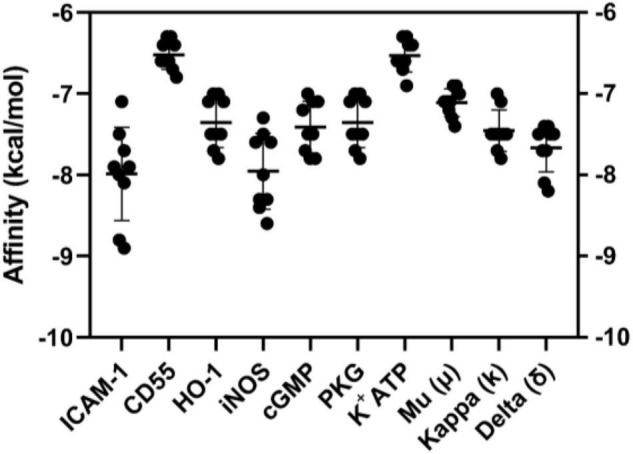
Graphical representation (means ± SD) of binding energy values of molecular dockings between MC-H and ICAM-1, CD55, HO-1, iNOS, soluble cGMP, cGMP-dependent protein kinase (PKG), K^+^_ATP_ channel, mu (μ), kappa (k), and delta (δ) opioid receptors. AutoDock Vina was employed to calculate the affinity values demonstrated.

The interactions of MC-H with the targets of interest were tested *in silico* by molecular docking. Figure 1 depicts the affinity (kcal/mol) of MC-H in all simulations. All interactions presented acceptable binding energy values, below −6.0 kcal/mol, which suggest MC-H might strongly bind to the molecular targets of interest. Mean values of binding energy in kcal/mol (± standard deviation) of MC-H were determined for each potential target as follows: ICAM-1 (−8.0 ± 1.10 kcal/mol), CD55 (−6.6 ± 0.20 kcal/mol), HO-1 (−7.4 ± 0.82 kcal/mol), iNOS (−7.9 ± 1.02 kcal/mol), soluble cGMP (−7.5 ± 0.74 kcal/mol), cGMP-dependent protein kinase (PKG) (−7.4 ± 0.82 kcal/mol), K^+^_ATP_ channel (−6.6 ± 0.38 kcal/mol), mu (μ) opioid receptor (−7.1 ± 0.45 kcal/mol), kappa (k) opioid receptor (−7.6 ± 0.81 kcal/mol), and delta (δ) opioid receptor (−7.7 ± 0.68 kcal/mol).

**FIGURE 2:**
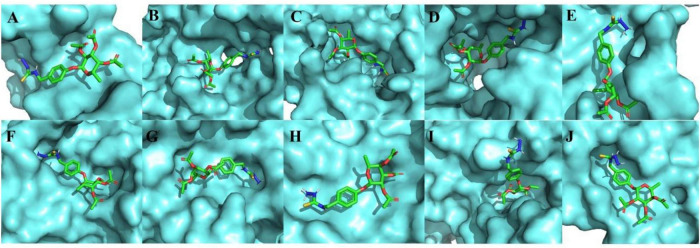
Three-dimensional representation of molecular dockings between MC-H and the molecular targets of interest. **(A)** ICAM-1, **(B)** CD55, **(C)** HO-1, **(D)** iNOS, **(E)** soluble cGMP, **(F)** cGMP-dependent protein kinase (PKG), **(G)** K^+^_ATP_ channel, **(H)** mu (μ) opioid receptor, **(I)** kappa (k) opioid receptor, and **(J)** delta (δ) opioid receptor.

Furthermore, the three-dimensional structure of molecular dockings between MC-H at different binding sites of the molecular targets of interest is shown in Figure 2. The MC-H structure presented a flexible behavior with several interactions with azanide regions, strong hydrogen interactions, and many hydrophobic bonds in all interactions tested. A re-docking was performed to compare native ligands of the regions with which MC-H interacts. Thus, confirming in all structures the correct interaction of MC-H within the pocket of each molecular target.

### MC-H Down-Regulates the Levels of Intercellular Adhesion Molecule (ICAM-1) and Decay-Accelerating Factor (CD55) in Formalin-Induced Hypernociception in the TMJ

**FIGURE 3:**
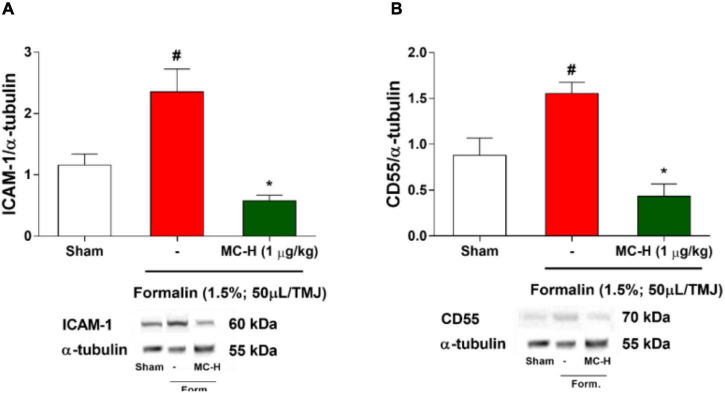
**(A)** ICAM-1 and **(B)** CD55 levels in the periarticular tissue after formalin-induced TMJ nociception. Administration of MC-H (1 μg/kg; *po*) significantly decreased the levels of both ICAM **(A)** and CD55 **(B)**, which were significantly lower than the formalin group. Values are expressed as mean ± SEM for all groups. The symbols (#) and (*) represent statistically significant differences compared with sham and formalin groups, respectively (*p* < 0.05: ANOVA, Tukey’s test). Representative ICAM-1, CD55, and GAPDH bands of each group are displayed below the graph.

Western blotting analysis demonstrated that intra-articular injection of formalin solution (1.5%; 50 μl/TMJ) increased ICAM-1 levels in the periarticular tissue when compared with the sham group as shown in Figure 3A. The same characteristic effect was observed concerning the CD55 levels as shown in Figure 3B. The treatment with MC-H (1 μg/kg; *po*), 60 min before the intra-articular injection of formalin, significantly reduced the levels of both ICAM-1 and CD55 in the TMJ periarticular tissue (*p* < 0.05) (Figures 3A,B).

### HO-1 Pathway Mediated the Antinociceptive Effects of MC-H

**FIGURE 4:**
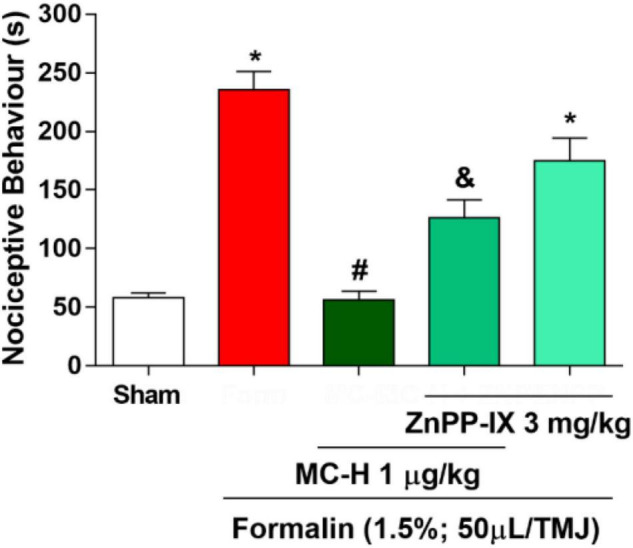
MC-H-induced antinociception depends on heme oxygenase-1 (HO-1) integrity. Administration of MC-H (1 μg/kg; *po*) significantly decreased the nociceptive behavior induced by formalin in the TMJ. Pre-treatment with ZnPP IX (3 mg/kg), an HO-1 inhibitor, significantly decreased the antinociceptive effects of MC-H. The symbols (*), (#), and (&) represent statistically significant differences compared with saline, formalin, and MC-H groups, respectively (*p* < 0.05: ANOVA, Tukey’s test).

Figure 4 shows that the intra-articular injection of the formalin solution (1.5%; 50 μl/TMJ) significantly increased the nociceptive behavior when compared to the sham group, as well as the MC-H (1 μg/kg; *po*) reduced the nociceptive behavior when compare to the formalin group. The pre-treatment with ZnPP-IX (3 mg/kg; *sc*), a specific HO-1 inhibitor, partially reversed the antinociceptive effect of MC-H (1 μg/kg; *po*) in the formalin-induced TMJ hypernociception (*p* < 0.05), and ZnPP when applied previously to the formalin into the TMJ without MC-H was similar to the formalin group, showing no effect of this pharmacological agent alone. This result suggests that the antinociceptive effect of MC-H in part depends on the HO-1 induction.

### Involvement of the NO/cGMP/PKG/K^+^_ATP_ Pathway in the Antinociceptive Effect of MC-H

**FIGURE 5:**
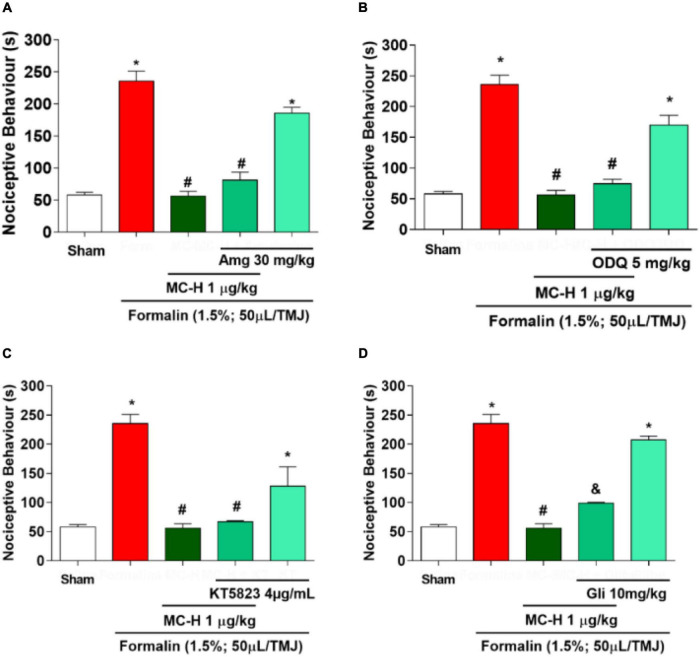
The role of NO/cGMP/PKG/K^+^_ATP_ signaling pathway in the MC-H-induced antinociception. Rats received the NO/cGMP/PKG/K^+^_ATP_ pathway inhibitors 30 minutes before MC-H (1μg/kg; po) treatment. The nociceptive behavior was evaluated 1 h after the MC-H administration. **(A)** Aminoguanidine - Amg (30 mg/kg; *ip*), **(B)** ODQ (5 mg/kg; *sc*) or **(C)** KT5823 (4 μg/mL; *sc*) did not modify the nociceptive response of MC-H (*p* < 0.05: ANOVA, Tukey’s test). However, **(D)** the pre-treatment with glibenclamide - Gli (10 mg/kg; *ip*) increased the nociceptive behavior (*p* < 0.05: ANOVA, Games-Howell test). The symbols (*), (#), and (&) represent statistically significant differences compared with saline, formalin, and MC-H groups, respectively.

The intra-articular injection of the formalin solution (1.5%; 50 μl/TMJ) significantly increased the nociceptive behavior when compared to the sham group, as well as the MC-H (1 μg/kg; *po*) reduced the nociceptive behavior when compared to the formalin group. The injection of aminoguanidine, an iNOS inhibitor, or ODQ, a specific inhibitor of the soluble cGMP, or KT5823, a selective inhibitor of cGMP-dependent protein kinase (PKG), did not reverse the antinociceptive effect of MC-H in the formalin-induced TMJ nociception, whereas the K^+^_ATP_ channel blocker glibenclamide prevented the MC-H- mediated analgesic effect (*p* < 0.05). Also, when these pharmacological agents were applied previously to the formalin into the TMJ without MC-H, the nociceptive behavior was similar to the formalin group, showing no effect of them alone (Figure 5).

### MC-H Inhibits Formalin-Induced Nociception in the Temporomandibular Joint via Central Opioid System Activation

**FIGURE 6:**
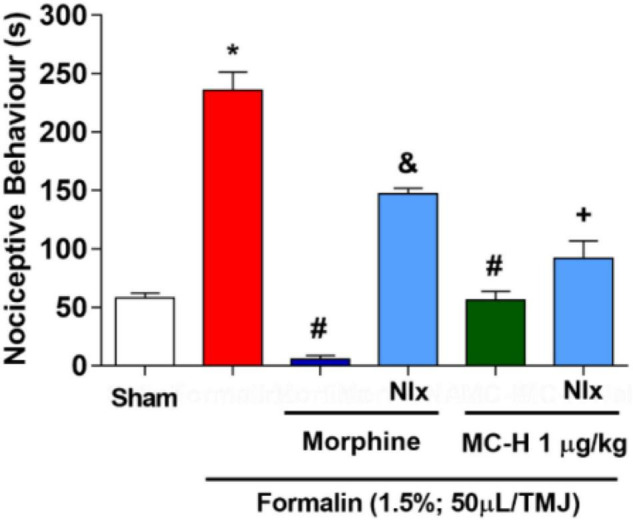
MC-H-induced antinociception depends on central opioids receptors. Administration of MC-H (1 μg/kg; *po*) significantly decreased the nociceptive behavior induced by 1.5% formalin in the TMJ. Pre-treatment of naloxone (Nlx), a non-selective opioid antagonist, affected the antinociception induced by MC-H. The symbols (*), (#), (&), and (+) represent statistically significant differences compared with saline, formalin, morphine, and MC-H groups, respectively. (*p* < 0.05: ANOVA, Games-Howell test).

**FIGURE 7:**
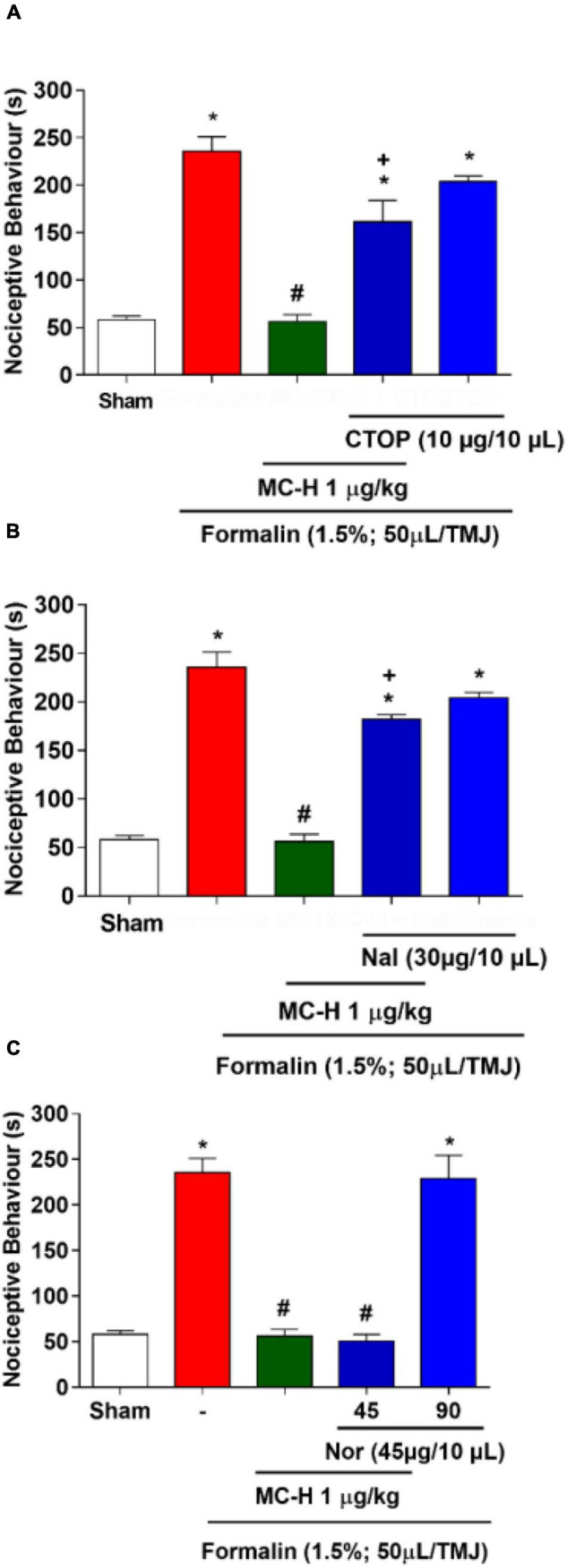
MC-H-induced antinociception depends on central μ- and δ-opioid receptors. Intrathecal administration of **(A)** CTOP (10 μg/μL), a selective μ-opioid receptor antagonist, or **(B)** naltrindole, Nal (30 μg/10 μL), a δ-opioid receptor antagonist, abolished the antinociception induced by MC-H. **(C)** Norbinaltorphimine, Nor (45 μg/10 μL), a selective κ-opioid receptor antagonist, did not inhibit the MC-H-induced antinociception. The symbols (*), (#), and (+) represent statistically significant differences compared with saline, formalin, and MC-H groups, respectively, (*p* < 0.05: ANOVA, Games-Howell test).

The intra-articular injection of the formalin solution (1.5%; 50 μl/TMJ) significantly increased the nociceptive behavior when compared to the sham group, as well as morphine and the MC-H (1 μg/kg; *po*) reduced the nociceptive behavior when compared to the formalin group. The sub-arachnoid administration of naloxone prevented (*p* < 0.05) the antinociceptive effect for the both of morphine and MC-H (1 μg/kg; *po*) as shown in Figure 6. This indicated that there might be a correlation between the activation of central opioid receptors and the antinociceptive effect of MC-H. To investigate it in more detail, the blockade with μ, κ, and δ opioid receptor-specific antagonists were also performed. Figures 7A,B show that central administration of selective μ-opioid receptor antagonist CTOP (10 μg/μL) and the selective δ-opioid receptor antagonist naltrindole (30 μg/10 μL) abolished the antinociceptive effect of MC-H (*p* < 0.05). Despite this, the intrathecal injection of norbinaltorphimine (45 μg/10 μL), a selective κ-opioid receptor antagonist, did not inhibit MC-H-induced antinociception as shown in Figure 7C (*p* > 0.05). Also, when these pharmacological agents were applied previously to the formalin into the TMJ without MC-H, the nociceptive behavior was similar to the formalin group, showing no effect of them alone (Figure 7).

## Discussion

The semisynthetic derivative MC-H is a hydrazine carbothioamide obtained from the benzylisothiocyanate-type natural product (MC-1) isolated as main constituent from flowers of *M. oleifera*. Previously, our research group showed that MC-H was not toxic when applied in mice by gavage in daily doses for 14 days (dos Santos et al., 2018). In the present study, molecular docking simulation was carried out with the targets of interest [ICAM-1, CD55, HO-1, iNOS, soluble cGMP, cGMP-dependent protein kinase (PKG), K^+^_ATP_ channel, mu (μ), kappa (k), and delta (δ) opioid receptors] and all of them showed high binding affinity to the binding site of MC-H. These data were confirmed by *in vivo* assays showing that the MC-H mechanism of action involves, at least in part, downregulation of ICAM-1 and CD55 levels in TMJ periarticular tissues. Furthermore, the mechanisms that mediate MC-H antinociception can also be explained in part by peripheral HO-1 activity and activation of central opioid receptors. Also, molecular docking has a satisfactory consistency and represents a complementary method for drug development, and it should be used together with *in vitro* and *in vivo* studies. In the present study we found a satisfactory correlation between *in silico* docking studies and *in vivo* activity. Considering the 10 targets of interest that showed high binding affinity to the binding site of MC-H in the docking studies, six of them were confirmed by the *in vivo* tests.

MC-H is a promising molecule showing high antinociceptive effects even at low concentrations, showing the same effect of indomethacin and morphine in a 5,000 times lower dose (dos Santos et al., 2018). This occurs possibly due to the triple acetylation at the carbons (2′, 3′, and 4′) of the rhamnose sugar provides more stability and renders the molecule more lipophilic, enabling a greater ability to cross organic barriers such as the blood-brain barrier (Dvorak, 2010; do Val et al., 2018). Tests performed with seven semi-synthetic compounds produced from MC-1 isolated from *M. oleifera* and structurally similar to MC-H (presence of benzene ring and sugar rhamnose) showed that the anti-inflammatory activity of these compounds is reduced by up to 8 times in un-acetylated derivatives (dos Santos et al., 2018).

In nature, glucosinolates produce isothiocyanates after the disruption of the plant tissue and activation with the enzyme myrosinase. Plants produce isothiocyanates with the main function of protection against insects and microbial invaders (Dinkova-Kostova and Kostov, 2012; Galuppo et al., 2015). In addition to plants, the human, mouse, and rat gut microflora can also breakdown glucosinolates into isothiocyanates (Rouzaud et al., 2003; Zhu et al., 2010; Wu et al., 2019). However, recent studies have shown that these compounds may exhibit various biological activities in animal cells, including anticancer, chemopreventive, hypoglycemic, antioxidant, analgesic and anti-inflammatory effects (Dhakad et al., 2019; Lopez-Rodriguez et al., 2020; Wu et al., 2021). In addition, there is evidence that isothiocyanates act to protect against neurodegenerative and cardiovascular diseases since they have the ability to produce varied and long-lasting responses that defend against oxidative stress, electrophilic stress, chronic inflammation, and apoptosis (Brunelli et al., 2010; Kamal et al., 2022). Different from those present in cruciferous vegetables, like broccoli and watercress, the isothiocyanates from *M. oleifera* have higher chemical stability due to the presence of an additional sugar bound to a benzene ring. In the case of MC-1, this sugar is the rhamnose monoacetylated in 4′-position, and which is hyperacetylated in the MC-H molecule after its semi-synthesis. These chemical characteristics permit the compound to be solid, odorless, and relatively stable at room temperature (Waterman et al., 2014).

The formalin-induced TMJ hypernociception is a validated model widely used in the literature for the experimental study of deep face pain. Formalin evokes a nociceptive response in two distinct phases. First, serotonin and histamine are released at the inflammatory site, acting directly on nociceptors of C- and A-delta-type fibers. In the second phase, prostaglandins, cytokines, and sympathomimetic amines mediate the previous sensitization of nociceptors (Jiménez et al., 2006; Chicre-Alcântara et al., 2012). However, when applied to the TMJ, formalin induces a single-phase nociceptive behavior of rubbing the orofacial region and flinching the head quickly (Roveroni et al., 2001; Fischer et al., 2005). Considering our previous result showing that MC-H (1 μg/kg; *po*) was the most efficient dose in the experimental model of TMJ hypernociception, this dose was used as a standard *in vivo* assays to determine the MC-H mechanisms of action.

Plasma extravasation promotes the passage of proteins and leukocytes into tissues and it is, therefore, considered a major sign of inflammation (Yang et al., 2005; Silva Quinteiro et al., 2014). In the Evans Blue Dye plasma extravasation test, MC-H reduced the amount of protein extravasation into the TMJ periarticular tissue (dos Santos et al., 2018), suggesting a potent anti-inflammatory effect. One of the first steps in the inflammatory process is the migration of circulating leukocytes to tissues. There is a cascade of events that commands leukocyte recruitment, namely rolling, firm adhesion, and, ultimately, transmigration. For this purpose, cellular binding is mediated by activation of adhesion receptors in leukocytes, followed by binding to counter-receptors in endothelial cells. The endothelial intracellular adhesion molecule-1 (ICAM-1) interacts with the CD11/CD18 complex of leukocytes and mediates transmigration. On the other hand, the decay-accelerating factor (DAF, also called CD55) functions as an anti-adhesive molecule that promotes the clearance of epithelial-bound leukocytes (Karpus et al., 2015).

We have shown that MC-H prevented an increase of formalin-induced ICAM-1 in periarticular tissues. Also, the amount of CD55 protein remained at baseline levels. It has been demonstrated that the induction of endothelial ICAM-1 is mediated by phospholipase A2α with the participation of the transcription factor NF-κB (Hadad et al., 2011). This suggests that the regulation of inflammatory factors may inhibit the production of ICAM-1. Similarly, the expression of the CD55 molecule is controlled by the hypoxia-induced factor (HIF), functioning as a protective mechanism (Louis et al., 2005). Therefore, it is likely that in the absence of pro-inflammatory factors, ICAM-1 levels are reduced and consequently avoid the increase in CD55 levels. However, more studies are needed to support this hypothesis.

It is known that glucosinolates and isothiocyanates derived from *M. oleifera* can decrease the gene expression and production of IL-1β, TNF-α, and nitric oxide synthase (iNOS) enzyme in RAW macrophages cells (Cheenpracha et al., 2010; Waterman et al., 2014; Giacoppo et al., 2017). These inflammatory mediators are primarily responsible for chemical and cellular changes in endothelium, injured tissue, and circulating cells that lead to increased vascular permeability and leukocyte migration (Tancharoen et al., 2008). Indeed, inflammatory pain in peripheral tissue depends on the activation of the TNF-α type 1 receptor in the primary afferent neuron, and, specifically in the TMJ, cytokines play a crucial role on pain and inflammation reaction (Kellesarian et al., 2016; Magalhães et al., 2021). TNF-α and IL-1β concentrations have been determined in the TMJ periarticular tissue and trigeminal ganglion associated with other nociceptive pathways as HO-1, NO and opioid (Chaves et al., 2011, 2018; Messlinger et al., 2020; Wang et al., 2021). The ability of isothiocyanates to inhibit these mediators appears to occur by blocking IκB-α phosphorylation and nuclear translocation of NF-κB, a transcription factor that regulates several pro-inflammatory genes (Giacoppo et al., 2017). Besides, animal model tests of multiple sclerosis, an inflammatory demyelinating disease in which vascular endothelium plays a primordial role, treated with glucomoringin isothiocyanates, confirmed that glucomoringin compound can neutralize the inflammatory cascade, promoting neuronal and axonal protection (Brunelli et al., 2010). This result was demonstrated by the reduction of TNF-α and iNOS levels and the improvement of histological parameters in spinal cord samples (Brunelli et al., 2010).

Heme oxygenase-1 (HO-1) is a cytoprotective enzyme induced at the site of inflammation and converts the heme group into carbon monoxide (CO), biliverdin, and free iron. Studies have reported linking activation of HO-1 with the prevention of peripheral neuropathic pain in rats (Bijjem et al., 2013). HO-1 is also present in the dorsal root ganglia, in the trigeminal ganglia, and in higher regions of the nociceptive system, exerting protective and adaptive mechanisms (Shen et al., 2015; Akram et al., 2016). We have previously demonstrated that HO-1 plays a role in the physiopathology of TMJ pain, exerting anti-inflammatory effects (Chaves et al., 2018). Since the HO-1 pathway appeared to be such a crucial factor, we raised the question of whether MC-H effects would depend on the integrity of this pathway. Our data suggested that peripheral HO-1 participates in the antinociceptive effect of MC-H, corroborating with the mentioned findings (Chaves et al., 2018).

The NO/cGMP/PKG/pathway did not appear to regulate the ability of MC-H to decrease peripheral neuronal sensitization since inhibition of nitric oxide synthase, guanylyl synthase, and protein kinase G did not change MC-H pre-treated animals. It has already been shown that systemic glibenclamide can cross the blood-brain barrier and reduce brain damage secondary to ischemic stroke (Khanna et al., 2014). This ability to cross organic barriers is potentiated by the presence of inflammation (Simard et al., 2012; Caffes et al., 2015). Once bound to the K^+^_ATP_ channel, glibenclamide prevents K^+^ efflux, reducing neuronal hyperpolarization and, consequently, decreasing neuronal firing (Nisticò et al., 2007). Therefore, glibenclamide (ip) may have centrally blocked the MC-H effects, where other NO pathway components do not lead to antinociception.

Central opioid receptors are involved in pain regulatory pathways and pharmacological studies have searched for drugs that act on these receptors for pain therapies (Stein and Machelska, 2011; Giri and Hruby, 2014; Linz et al., 2014). However, most drugs have severe adverse effects such as constipation, nausea, dependence, and other potentially dangerous conditions like respiratory depression (Manglik et al., 2016). There is evidence, both experimental and clinical, that some chronic pain conditions result from changes that reduce the effect of downstream modulating pathways (Sessle, 2011, 2021). Therefore, studies on drug design and the development of new compounds with activity on opioid receptors and fewer side-effects are highly necessary. Our data suggest that MC-H may act directly or indirectly on central opioid receptors with a partial selectivity for μ and δ-opioid receptors.

As a whole, we determined that MC-H efficacy in a pre-clinical study of TMJ pain is mediated, at least in part, peripherally by the HO-1 action, as well as through downregulation of ICAM-1 and CD55 levels, and centrally by activation of opioid receptors (μ and δ). A study using a semisynthetic compound that has already been shown to be safe in a pre-clinical trial, along with the possibilities offered by *in silico* docking study in combination with a pre-clinical trial investigating the mechanism of action of this new compound, may contribute to fuel the development of a possible future new drug with a good safety/efficacy ratio in the treatment of TMJ pain relief.

## Conclusion

Our data provide evidence that a semisynthetic derivative MC-H obtained from *Moringa oleifera* Lam. flowers presents potential antinociceptive and anti-inflammatory effects in the rat TMJ when administered orally. Primarily, this potential analgesic effect is mediated peripherally by the HO-1 action, as well as through inhibition of adhesion molecule levels, and centrally by activation of opioid receptors (μ and δ).

## Data Availability Statement

The datasets presented in this study can be found in online repositories. The names of the repository/repositories and accession number(s) can be found below: http://www.repositorio.ufc.br/handle/riufc/29478.

## Ethics Statement

The animal study was reviewed and approved by Animal Care Unit of the Federal University of Ceará (UFC - Fortaleza, Brazil) under registration number 03/2015.

## Author Contributions

FS, MB, JC-N, and HC conceived and designed the research. FS, FG, DV, HF, EA, DA, and DC conducted the experiments. HB and RJ performed the computational analyses. FB, JM, MS, GB, VP, and GC-F contributed to the new reagents or analytical tools. FS, FG, MB, and HC analyzed the data. FS, FG, MB, and HC wrote the manuscript. All authors read and approved the manuscript.

## Conflict of Interest

The authors declare that the research was conducted in the absence of any commercial or financial relationships that could be construed as a potential conflict of interest.

## Publisher’s Note

All claims expressed in this article are solely those of the authors and do not necessarily represent those of their affiliated organizations, or those of the publisher, the editors and the reviewers. Any product that may be evaluated in this article, or claim that may be made by its manufacturer, is not guaranteed or endorsed by the publisher.

## Footnotes

1
https://www.rcsb.org/

2
http://sts.bioe.uic.edu/castp/calculation.html
